# Building Trust in Ethnically Diverse Older Adults Using Technology-Based Physio-Feedback

**DOI:** 10.1093/geroni/igaa057.1851

**Published:** 2020-12-16

**Authors:** Ladda Thiamwong

**Affiliations:** College of Nursing, University of Central Florida, Orlando, Florida, United States

## Abstract

There is no research exploring how to build trust in the context of falls risk assessment and prevention. This study describes strategies to build trust in ethnically diverse older adults using technology-based physio-feedback from two studies. The technology includes a portable BTrackS balance plate and BTrackS Balance Software running on a computer device. Participants were provided instant playback showing their static balance performance with a scale from 0 to 100. Sixty-seven community-dwelling older adults participated in the first study, and 41 of them (61.2%) participated again in the second study using the same procedures. 70% were women, 43% were immigrants, 34% Hispanics, 15% African Americans, and 9% Asians. Three reasons for participation were reported: 1) specific objective feedback on the test results that supported by technology, 2) ability to record changes over time; and 3) ability to access the fall risk technology-based test at a place of their convenience. Part of a symposium sponsored by the International Aging and Migration Interest Group.

